# Insights into factors sustaining persistence of high malaria transmission in forested areas of sub-Saharan Africa: the case of Mvoua, South Cameroon

**DOI:** 10.1186/s13071-020-04525-0

**Published:** 2021-01-02

**Authors:** Dominique Mieguim Ngninpogni, Cyrille Ndo, Patrick Ntonga Akono, Anicet Nguemo, Amine Nguepi, Danale Rosine Metitsi, Jeannette Tombi, Parfait Awono-Ambene, Charles Félix Bilong Bilong

**Affiliations:** 1 Laboratory of Parasitology and Ecology, Faculty of Sciences, University of Yaoundé I, P.O. Box 812, Yaoundé, Cameroon; 2 Institut de Recherche de Yaoundé (IRY), Organisation de Coordination pour la lutte Contre les Endémies en Afrique Centrale (OCEAC), P.O. Box 288, Yaoundé, Cameroon; 3 Department of Biological Sciences, Faculty of Medicine and Pharmaceutical Sciences, University of Douala, P.O. Box 24157, Douala, Cameroon; 4 Department of Parasitology and Microbiology, Centre for Research in Infectious Diseases (CRID), P.O. Box 13591, Yaoundé, Cameroon; 5 Animal Organisms Laboratory, Faculty of Sciences, University of Douala, P.O. Box 24157, Douala, Cameroon

**Keywords:** Malaria prevalence, Insecticide resistance, *Anopheles*, Long lasting insecticide treated net, Mvoua

## Abstract

**Background:**

In Mvoua, a village situated in a forested area of Cameroon, recent studies have reported high prevalence of *Plasmodium falciparum* infection among the population. In order to understand factors that can sustain such a high malaria transmission, we investigated the biology of *Anopheles* vectors and its susceptibility to insecticides, as well as long-lasting insecticidal net (LLIN) coverage, use and bio-efficacy.

**Methods:**

A longitudinal entomological survey was conducted from July 2018 to April 2019. Adult mosquitoes were collected using the human landing catch (HLC) method and identified using morphological and molecular techniques. *Anopheles gambiae* (*s.l.*) larvae were sampled from several stagnant water pools throughout the village and reared to generate F1 adults. The presence of *P. falciparum* circumsporozoite antigen was detected in the heads and thoraces of mosquitoes collected as adults using an enzyme-linked immunosorbent assay. The insecticide susceptibility status of the local *An. gambiae* (*s.l.*) F1 population to the pyrethroid insecticides deltamethrin 0.5% and permethrin 0.75% was determined using World Health Organization-tube bioassays, while the frequency of the knockdown resistance (*kdr*) mutation was determined by PCR. Coverage, use and physical integrity of LLINs were assessed in households, then cone assays were used to test for their bio-efficacy on both the reference insecticide-susceptible Kisumu strain and on field F1 *An. gambiae* (*s.l.*)

**Results:**

In total, 110 *Anopheles* mosquitoes were collected, of which 59.1% were identified as *Anopheles funestus* (*s.l.*), 38.18% as *An. gambiae* (*s.l.*) and 2.72% as *An. ziemanii*. *Anopheles funestus* was the most abundant species except in the long rainy season, when *An. gambiae* (*s.l.*) predominated (65.8%). In the dry seasons, vectors were principally endophagous (76% of those collected indoors) while they tended to be exophagous (66% of those collected outdoors) in rainy seasons. High *Plasmodium* infection was observed in *An*. *gambiae* (*s.l.*) and *An. funestus*, with a circumsporozoitic rate of 14.29 and 10.77%, respectively. *Anopheles gambiae *(*s.l.*) was highly resistant to pyrethroid insecticides (mortality rates: 32% for permethrin and 5% for deltamethrin) and harbored the* kdr*-L1014F mutation at a high frequency (89.74%). Of the 80 households surveyed, only 47.69% had achieved universal coverage with LLNs. Around 70% of the LLINs sampled were in poor physical condition, with a proportionate hole index > 300. Of the ten LLNs tested, eight were effective against the *An. gambiae* reference insecticide-susceptible Kisumu strain, showing mortality rate of > 80%, while none of these LLINs were efficient against local *An. gamabie* (*s.l.*) populations (mortality rates < 11.5%).

**Conclusion:**

A combination of elevated *P. falciparum* infection in *Anopheles* vector populations, insufficient coverage and loss of effectiveness of LLINs due to physical degradation, as well as high resistance to pyrethroid insecticides is responsible for the persistence of high malaria transmission in forested rural area of Mvoua, Cameroon. 
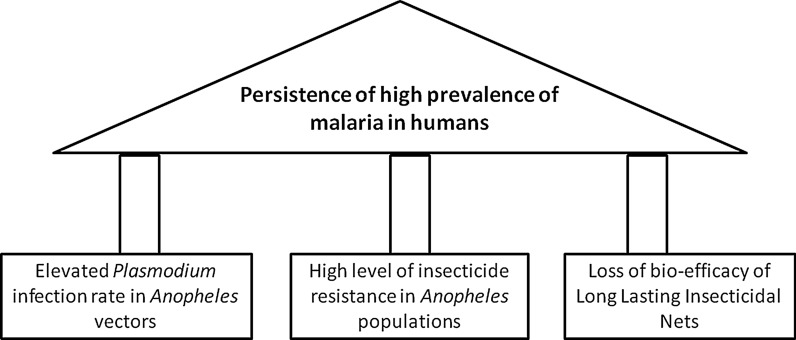

## Background

Malaria remains the most endemic parasitic disease worldwide. The African continent is by far the most malaria-affected region, with approximately 93% of all cases (213 million) and 94% of deaths worldwide [1]. In Cameroon, malaria is highly endemic and represents an important health threat, particularly for the most vulnerable groups, including children aged < 5 years, pregnant women and people living with human immunodeficiency virus/acquired immunodeficiency syndrome [2]. The epidemiology of malaria is heterogeneous throughout the country, with high and perennial transmission occurring in the forest, coastal and humid savanna areas and low transmission in the highlands, while the Sahel and dry savanna regions are areas of seasonal transmission. Whatever the area, the disease is mainly caused by the *Plasmodium falciparum* parasite, with mosquitoes from the *Anopheles gambiae* complex and *An. funestus* group being its major vectors [3]. Both species have wide geographic distributions and co-exist over much of their range. Their efficiency as malaria vectors is due to their high dependence on humans for their feeding, resting, and to a certain extent, breeding habitats [4].

The fight against malaria relies mainly on vector control through the use of long-lasting insecticidal nets (LLINs) and indoor residual spraying of insecticides. To this end, more than 20 million of LLINs have been distributed to the Cameroonian population since 2011. This scale-up of LLINs across the country resulted in a significant decrease in reported malaria cases, from 41 to 24.3%, between 2000 and 2015. During this same time span, it was estimated that there was a 54% decrease in malaria-related mortality, from about 13,000 to 6000 deaths per year. However, despite these gains, parasitological and entomological studies conducted in the rural settings of Cameroon still reveal high prevalences of *Plasmodium* infection in both the human and mosquito hosts. This is especially the case in the forested areas found in the southern part of the country. Such high prevalences bring into question the effectiveness of the LLINs distributed as well as possible changes in vector biology [5–7].

Effective malaria vector control is reliant on knowledge of the local vector species and their susceptibility to insecticides. Generally, within a given area, the biology of each species is unique in many respects, including the sites where larvae develop, adult mosquito behavior (especially human-biting behavior), susceptibility to *Plasmodium* parasites, the ability to transmit these parasites and their resistance profile to insecticides [8]. Therefore, a failure to appreciate the biological complexities that allow vector populations to sustain malaria transmission and to resist or evade interventions could substantially impede control efforts [9]. Moreover, there is also a critical need for ongoing monitoring of the coverage, usage, quality and durability of vector-control interventions following their deployment. Periodic collection of such data is essential to inform vector control strategies and track their impact on malaria transmission [9, 10].

The present study was conducted in Mvoua, a village situated in the forested region of Cameroon where high malaria prevalence has been reported [7]. Preliminary investigations have been carried out at this site previously, with the aim to understand factors contributing to such a high prevalence by assessing population knowledge, attitudes and practices related to malaria transmission and disease case management. In the study reported here, we investigated other key factors which could influence malaria epidemiology, such as *Anopheles* biology and susceptibility to insecticides, and LLIN coverage, use and effectiveness, with the aim to contribute to malaria surveillance in Cameroon [11, 12].

## Methods

### Study area

The study was conducted in Mvoua (4°4′N; 11°25′E), a village situated in Okola district in the Central region of Cameroon. This village of about 300 inhabitants is located approximately 35 km from Yaoundé, the political capital of the Republic of Cameroon. The vegetation cover is made up of a semi-deciduous forest subjected to a Guinean-equatorial climate type, with mean annual rainfalls ranging from 1600 to 1800 mm. This climate is characterized by two distinct rainy seasons extending from March to May (short rainy season) and from August to November (long rainy season), and by two dry seasons extending from June to July (short dry season) and from December to February (long dry season) [13]. A previous malaria diagnosis survey conducted in Mvoua reported that malaria remains highly endemic in this locality, with infection prevalence of 60% in children aged between 4 and 10 years [7].

### Adult mosquito collection

Collections of adult host-seeking mosquitoes were undertaken employing both the CDC light trap (CDC-LT) and human landing catch (HLC) methods. Because no mosquito was collected using the CDC-LTs after two consecutive nights of sampling, this method was discarded.

Sampling using the HLC method was performed in ten randomly selected houses situated at least 100 m apart. The method involved persons sitting with their lower legs exposed and then collecting mosquitoes that just landed on them [14]. Adult mosquitoes were sampled both indoors and outdoors during two consecutive nights, once per each of the four seasons. Mosquitoes collected each hour were placed into separate bags, labeled accordingly and brought back to the laboratory for further analysis. To avoid bias due to sleep and tiredness, one team of collectors worked from 18:00 h to midnight, and was replaced by another team which worked from midnight to 6:00 h.

### Mosquito identification and detection of *Plasmodium* infection

Adult mosquitoes collected using the HLC method were identified based on morphological criteria following the identification keys of Gillies and De Meillon [15] and Gillies and Coetzee [16]. Female *Anopheles* mosquitoes were sorted from other *Culicinae*, stored in Eppendorf tubes with silica gel (desiccant) and taken to the medical entomology laboratory of OCEAC (Organisation de Coordination pour la lutte Contre les Endémies en Afrique Centrale) for subsequent analyses. Heads and thoraces were processed for detection of *P. falciparum* circumsporozoite protein (CSP) using an enzyme-linked immunosorbent assay (ELISA) method as previously described [17, 18]. DNA extracted from the abdomen and legs [19] was used for the molecular identification of sibling species by PCR, as described previously [20, 21].

### Insecticide bioassays and knockdown resistance detection

Larvae of *An. gambiae* (*s.l.*) were collected in stagnant pools of water and reared in the insectary of the medical entomology laboratory of OCEAC. F1 adults that emerged from those larvae were fed with 10% sucrose solution made by dissolving 100 g of ordinary white sugar in 1 L of water [22]. *Anopheles funestus* was not tested due to the low number of adults obtained following larval collections and rearing. The susceptibility of the F1 adults to 0.75% permethrin and 0.05% deltamethrin, both pyrethroid pesticides, was assessed using a World Health Organization (WHO) standard test procedure [23]. Tests were performed at 25 ± 2 ℃, 80% ± 10% relative humidity (RH). For each insecticide, four batches of 20–25 field F1 females, aged between 2 and 5 days, were exposed to insecticide-impregnated papers in WHO test-tube for 1 h. At the same time, two batches of the same number of mosquitoes were exposed to untreated papers as control. At the end of the insecticide exposure period, the number of knocked-down mosquitoes was recorded, following which the mosquitoes were transferred into holding tubes. Cotton balls that had first been soaked in a 10% sugar solution and then the moisture squeezed out were placed at the mouth of the tubes. The mortality was recorded 24 h later. Mortality rate in the tested samples was corrected using Abbott’s formula [24], when the mortality in the control tubes varied between 5 to 20%. The knockdown resistance (*kdr*) mutation L1014F, which is responsible of cross resistance to DDT (dichlorodiphenyltrichloroethane) and pyrethroids was genotyped using the protocol described by Martinez-Tores et al. [25].

### Bed net coverage, use and maintenance

Bed net ownership was investigated in 80 of 97 the households that currently make up the village through visual inspection. The heads of these households or their representatives were questioned on their use of nets and their maintenance. The number of people living in each house was recorded to estimate net coverage. Household net ownership was defined as the percentage of households owning at least one LLIN. Net coverage defined as the percentage of households with at least one LLIN for every two people was also determined.

### Bed net integrity and bio-efficacy

Ten bed nets were randomly collected from ten selected houses and immediately replaced by new ones. The physical integrity of the nets collected was determined by counting, per category, the number of holes that were approximately the size of a person’s thumb, fist or head, or larger than a head, on any of the four faces and the top of the bed net [26, 27]. The proportionate hole index (pHI) was calculated using the WHO Pesticide Evaluation Scheme (WHOPES ) guidelines [11] and nets were classified into four classes accordingly [28, 29]. Nets with a pHI < 25 were classified as “good”; those with a pHI ranging between 25 and 174, as “fair”; those nets with a pHI ranging from 175 to 299, as “mediocre”; and those with a pHI > 300, as “poor”.

Cone assays were performed on each net to test for its bio-efficacy as described in WHO guidelines [30]. Four cones were fixed by their widest opening onto four different parts of each face (4 sides and 1 roof) of each net. Ten unfed *An. gambiae* (*s.l.*) female mosquitoes aged 2–5 days were introduced into each cone using a mouth aspirator, for a total of 200 specimens per net. After 3 min of exposure, the mosquitoes were removed from the cones, then transferred into paper cups and fed with a 10% sugar solution. The assay was conducted at 25 ± 1 ℃, 80% ± 5% RH, and the number of mosquitoes knocked-down and dead were recorded after 60 min and 24 h, respectively. An untreated net was used as the negative control.

### Data analysis

Data were analyzed using SPSS software version 25 (IBM Corp., Armonk, NY, USA). The entomological parameters that were considered were: (i) human-biting rate (HBR), i.e. the average number of bites received per person per night; (ii) infection rate, i.e. the proportion of mosquitoes found with *Plasmodium* circumsporozoite antigen in the heads and thoraces; (iii) entomological inoculation rate (EIR), i.e. the product of the HBR and circumsporozoite rate. Chi-square statistics was used to compare mosquito densities between seasons while mosquitoes’ seasonal aggressiveness (HBR) were compared by the Kruskal-Wallis test. Differences were considered statistically significant at* P* < 0.05.

## Results

### Composition and abundance of *Anopheles* mosquitoes

In total, 129 adult mosquitoes were collected during eight nights. These belonged to four genera, of which *Anopheles* (*N* = 110; 85.27%) was the most prevalent, followed in decreasing order of prevalence by *Mansonia* (*N* = 11; 8.53%), *Aedes* (*N* = 6; 4.65%) and *Culex* (*N* = 2; 1.55%). Three *Anopheles* species were collected namely *An. funestus* (*s.l.*) (59.1%), *An. gambiae* (*s.l.*) (38.18%) and *An. ziemanii* (2.72%). *Anopheles. sp* were most abundant in the short dry season followed by the long rainy season (49.09% of the total *Anopheles* collected) and the long rainy season (34.54%).

**Fig. 1.**
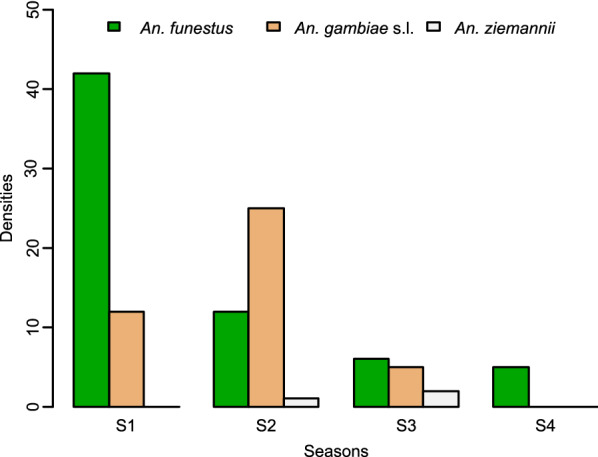
Seasonal relative abundance of* Anopheles* mosquito species in Mvoua. *S1* short dry season,* S2* long rainy season,* S3* long dry season,* S4* short rainy season

In terms of seasonal species abundance, *An. funestus* was the most abundant species collected (59.1%) over the period of study, with the exception of the long rainy season when *An. gambiae* (*s.l.*) predominated (Fig. 1).

### Entomological parameters

**Fig. 2:**
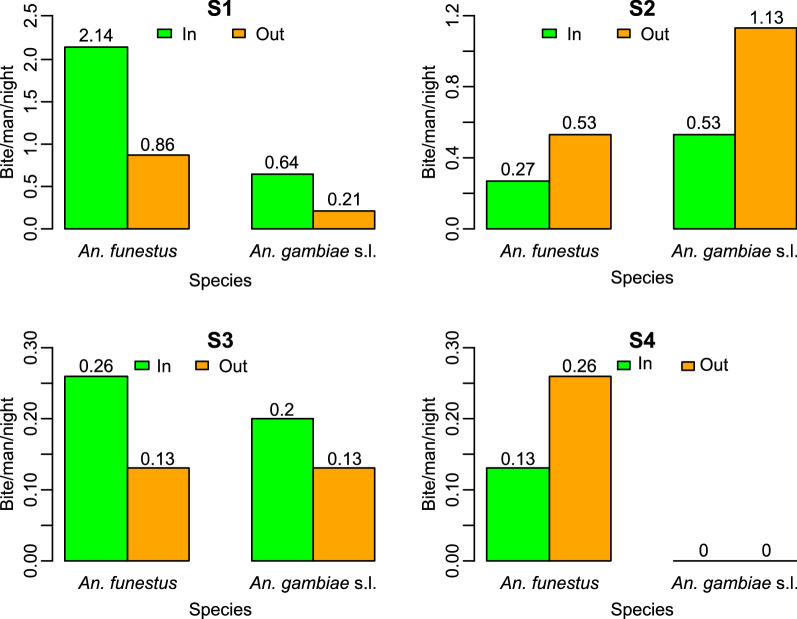
Seasonal variation in human biting behavior of malaria vectors in Mvoua from August 2018 to April 2019.* In* Inside (homes),* Out* outside, S1–S4 as defined in Fig. 1

The HBR was low during the entire study period, with the number of bites per person per night being less than one bite (0.93) (Table 1). The HBR of *An. funestus* (*s.l.*) was higher (0.55) than that of *An. gambiae* (*s.l.*) (0.35) (χ^2^ = 7.520, *df* = 1, *P* < 0.05). In terms of season and irrespective of the species, the difference in HBR was statistically significant (χ^2^ = 2.58, *df* = 3, *P* < 0.05), with the highest HBR value (1.92) observed during the short dry season and the lowest HBR value (0.17) observed during the short rainy season. *Anopheles funestus* (*s.l.*) was recorded as being the most aggressive (HBR 1.5) mosquito species during the short dry season, whereas *An. gambiae* (*s.l.*) had the higher HBR during the long rainy season (HBR: 0.83). During the two dry seasons, these two vectors were principally caught indoors (76%), while in rainy seasons, they were mostly found biting outdoors (66%) (Fig. 2).

**Table 1:** *Plasmodium* circumsporozoite index, human biting rate and monthly entomological inoculation rate of *Anopheles* species in Mvoua between August 2018 and April 2019

*Anopheles* species	Tested (*N*)	Positive (*N*)	Entomological parameters		
			*Plasmodium* circumsporozoite index (%)	Human biting rate	Entomological inoculation rate/month
*An. funestus* (*s.l.*)	65	7	10.77	0.55	1.5
*An. gambiae* (*s.l.*)	42	6	14.29	0.35	1.2
*An. ziemanii*	3	0	0	0.02	NC
Total	110	13	11.81%	0.93	2.7

Human-biting rate (HBR) is the average number of bites received per person per night; (ii)* Plasmodium* circumsporozoite index (infection rate) is the proportion of mosquitoes found with *Plasmodium* circumsporozoite antigen in the heads and thoraces; entomological inoculation rate is the product of the HBR and circumsporozoite rate

*NC* not calculated

**Fig. 3:**
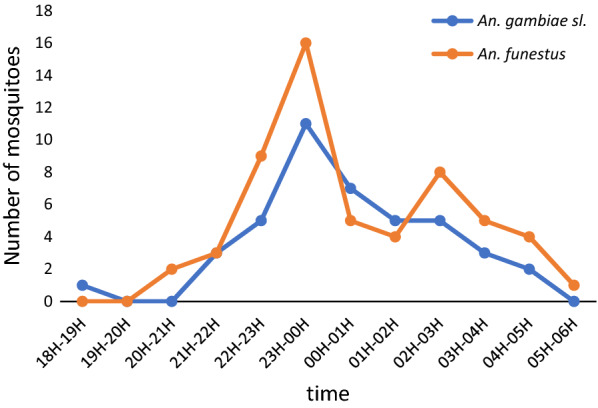
*Anopheles funestus* and *An. gambiae* (*s.l.*) night-biting cycle in Mvoua from August 2018 to April 2019

During the study period, most *Anopheles* bites from *An. funestus* (*s.l.*) and *An. gambiae* (*s.l.*) occurred between 21:00 h and 3:00 h, with the major peak being observed between 23:00 h and 00:00 h and the minor peak observed between 2:00 h and 3:00 h (Fig. 3).

### Mosquito infection and malaria transmission

Of the 110 samples tested, 13 (11.81%) were infected by *P falciparum* (Table 1). The circumsporozoite index (ICSP) was 10.77% for *An. funestus* (*s.l.*) and 14.29% for *An. gambiae* (*s.l.*), and both ICSP were not statistically different (χ2 = 0.296, *df* = 1, *P* > 0.05). The short dry season appeared to be the period of the year when the infection rate was the highest (ICSP 18.51%; χ2 = 3.86, *df* = 3, *P* < 0.05).

The overall EIR during the study was 2.7 infective bites/human/month, and there was no significant difference between the monthly EIRs of *An. funestus* (*s.l.*) (1.5 infective bites/human/month) and *An. gambiae* (*s.l.*) (1.2 infective bites/human/month) (χ2 = 8.54; *df* = 11, *P* > 0.05) (Table 1).

### Mosquito susceptibility to permethrin and deltamethrin

A total of 100 *An. gambiae* (*s.l.*) mosquitoes were exposed to each of the insecticides namely permethrin and deltamethrin. At the end of the exposure period (1 h), 32 and 5% mosquitoes were knocked-down for deltamethrin and permethrin, respectively. Low mortality, indicating a high level of resistance, was recorded for both insecticides, with a mortality rate of 33% for permethrin and 5% for deltamethrin. By contrast and as expected, the reference insecticide susceptible Kisumu strain used as control was fully susceptible to both insecticides (100% mortality).

The molecular studies performed on adult mosquitoes collected using the HLC method showed *An. funestus* (*s.s*) is the only member of the *An. funestus* group present in Mvoua. For the *An. gambiae* complex, *An. gambiae* was the most abundant species (71.8%) compared to *An. coluzzii* (28.2%). The *kdr* L1014F mutation was found to be present at high frequencies in both *An. gambiae* (98.21%) and *An. coluzzii* (77.27%) (Table 2).

**Table 2:** *Anopheles gambiae* complex species composition and the frequency of the knockdown resistance L1014F mutation

*Anopheles* species	Composition		*kdr*-L1014 F genotypes			*kdr*-L1014 F alleles (%)	
	*N*	%	RR	RS	SS	R	S
*An. gambiae*	28	71.8	27	1	0	98.21	1.79
*An. coluzzii*	11	28.2	8	1	2	77.27	22.73
Total	39	100	35	2	2	92.31	7.69

*kdr*-L1014F, Knockdown resistance L1014F mutation; R resistance; S, susceptible

### Possession, coverage and use of LLINs

In general, 81.25% of the households inspected possessed at least one LLIN. Despite this high level of ownership, the proportion of households that met the ratio of one net for two persons was only 47.69%. Fortunately, 81.54% of the people interviewed affirmed they used their bed nets every night (Table 3). Of the LLINs available, 92.3% were acquired during the government free mass distribution campaigns of 2011–2012 and 2015–2016. Although almost 70% of the study population affirmed that they have been educated by the public health community agents during the distribution campaigns, only 10.52% knew bed nets should be dried in the shade before usage or after washing. Moreover, 56.46% of respondents had washed their bet nets at least once; 63.15% used water and ordinary soap while 36.85% used detergents soap and bleach (Table 3).

**Table 3:** Origin, use and maintenance of long-lasting insecticidal nets

Variables related to LLIN	*N*	Frequency (%)
*Possession*		
Yes	65	81.25
No	15	18.75
*Origin*		
Government	60	92.3
Market	5	7.7
*Installed*		
Yes	59	90.76
No	6	9.24
*Presence per 2 persons*		
Yes	31	47.7
No	34	52.3
*Usage*		
Every night	53	81.54
Not every night	12	18.46
*Education received on LLINs during distribution*		
Yes	45	69.92
No	20	30.78
*Had LLIN been washed*		
Yes	38	58.46
No	27	41.54
*Substance used for washing*		
Ordinary soap	24	63.15
Detergent and bleach	14	36.85
*Drying place*		
In sun	34	89.48
In shade	4	10.52

LLIN, Long-lasting insecticidal net

### Physical integrity and effectiveness of LLINs

Ten LLINs aged 3–7 years old were sampled to assess their physical integrity and bio-efficacy. A total of 161 holes belonging to all the four different types described by WHOPES (the size of a person’s thumb, fist or head, or larger than a head) were counted, with a mean of 17 holes per bed net. The proportion of these holes per type was 52.79% (thumb size), 32.29% (fist size), 9.93% (head size) and 4.96% (large than head size). Of the ten LLINs examined, seven (70%) were damaged and were found to be in poor condition (pHI > 300) (Table 4).

**Table 4:** Hole index and mortality of *An. gambiae* (*s.l.*) after being exposed to the long-lasting insecticidal nets

Nets	Date of impregnation	Brand	Physical integrity of LLIN		Bio-efficacy of LLIN (%)		Decision
			pHI	Status	Kisumu control strain	Field strain	
Net 1	October 2015	Olyset	0	Good	83.50	3.50	Not efficient
Net 2	October 2015	Olyset	916	Poor	80.50	00.50	Not efficient
Net 3	February 2011	Permanet	1939	Poor	69.50	2.50	Not efficient
Net 4	February 2011	Permanet	581	Poor	88.00	11.00	Not efficient
Net 5	February 2011	Permanet	13	Good	99.00	8.00	Not efficient
Net 6	October 2015	Olyset	651	Poor	85.00	2.50	Not efficient
Net 7	October 2011	Permanet	2225	Poor	72.40	3.50	Not efficient
Net 8	October 2011	Permanet	1	Good	98.00	9.50	Not efficient
Net 9	May 2014	Royal sentry	1180	Poor	95.00	11.50	Not efficient
Net 10	October 2015	Olyset	1395	Poor	87.50	2.00	Not efficient

Not eff., not efficient; PHI, proportionate hole index

A total of 2000 specimens of the *An. gambiae* (*s.l.*) susceptible Kisumu strain were exposed to the ten bed nets. Eight of these nets were effective against this strain, with mortality rates > 80%. Exposure of the same number of *An. gambiae* (*s.l.*) field F1 mosquitoes to these LLINs revealed that they were all ineffective, with mortalities ranging from 0 to 11.5% (Table 4).

## Discussion

The results of this study show that the *Culicidae*-aggressive fauna in our study site consists of four genera, namely *Anopheles*,* Mansonia*,* Aedes* and *Culex*. The predominance of the *Anopheles* genus was likely due to our study locality being situated in a rural area. Indeed, several previous studies conducted in forested localities in Cameroon have reported the proliferation of mosquitoes of *Anopheles* genus in rural settings [31], while those of *Culex* and *Aedes* genera are predominant in highly anthropized and polluted urban areas [32]. Moreover, the use of the HLC method, which is a method for sampling anthropophilic mosquitoes, could have also favored the collection of athrophophilic *Anopheles* over other mosquito species [33–35]. However, while HLC remains the gold standard method for measuring human exposure to mosquito bites and, consequently, malaria transmission, it should be noted that results based on sampling using this method can be biased due to natural human variations in attractiveness to mosquitoes [36, 37].

Three main vector species belonging to the *An. funestus* group and *An. gambiae* complex were collected in this study. *Anopheles funestus* was more abundant throughout the study period, while two sibling species of the *An. gambiae* complex (*An. coluzzii* and *An. gambiae*) were mostly collected during the long rainy season. The pullulating period of *An. funestus* is usually observed at the end of the rainy season and up to the middle of the dry season [38, 39] while, in contrast, *An. gambiae* (*s.l.*) takes over as predominant species during the rainy seasons. It is well known that the intensity and frequency of rainfall during rainy seasons contribute greatly to the formation of temporary water pools, which represent typical breeding sites for *An. gambiae* (*s.l.*) [40, 41].

The values of the entomological parameters determined in our study further support findings from a previous parasitological survey conducted in Mvoua, also indicating the occurrence of high malaria transmission in this locality. The *Plasmodium* circumsporozoite rate determined by the ELISA was high in both vector species, confirming that mosquitoes from both the *An. funestus* group and *An. gambiae* complex are major vectors of malaria in the forested regions of Cameroon [42, 43]. Similar levels of *Plasmodium* infection have been recently reported by Ndo et al. [7] in Obout, another locality situated within the forested regions of central Cameroon, highlighting the persistence of high levels of malaria transmission in this environment, despite the intensive control efforts that have been deployed over the years. The forest environment actually offers suitable conditions for the proliferation of mosquito vectors throughout the year due to its climate, which is rainy, thereby conducive to the formation of temporary *Anopheles* breeding sites [44].

The high levels of infection in malaria vectors observed in this study are in striking contrast to the high possession rate of LLINs, which should have reduced malaria transmission by protecting individuals from infectious bites and by minimizing the probability that a mosquito survives throughout the entire extrinsic incubation period of the *Plasmodium* parasite. Our findings indicate rather that (i) *Anopheles* mosquitoes still have access to human hosts to ingest or transmit *Plasmodium* parasites during blood intake, and (ii) vectors still live long enough to enable the parasite to develop from the gametocyte to the infectious sporozoite stages. These findings therefore bring the efficiency of LLNs in the surveyed locality into question, and several observations from this study could help to elucidate this situation. Firstly, we observed an insufficient coverage in LLINs. Among the households inspected, although a high level of possession of LLINs was observed, the proportion of households that met the universal coverage as defined by WHO (one net for two persons) was still below 50%, and far from the targeted rate of 80% set by the Cameroonian government [45].

Secondly, LLINs were not optimally used. Although 81.54% of the respondents affirmed that they used their bed nets every night, it was noted that the use of LLINs varied depending of the season. A reduction in LLINs use is often observed during the dry season due to heat, and mosquitoes are more likely to migrate inside at this time, therefore increasing the probability of receiving infective bites [46]. In contrast, when the weather cools down, the proportion of people sleeping under bet nets scales up. The repellent effect of the nets could then drive mosquitoes outside, decreasing the probability of receiving an infective bite. Such behavior at the population level might have lead to a trend towards endophagy for *An. gambiae* (*s.l.*) and *An. funestus* (*s.s.*) during the two dry seasons, whereas they were more exophagous during the two rainy seasons. In addition, in our study, the short dry season appeared to be the period of the year when malaria transmission was higher.

Thirdly, LLINs could have lost their effectiveness due to physical degradation and decreased susceptibility of local vector populations to insecticides impregnated into the nets. The sampled LLINs were 3 to 7 years old, and 70% were found to be in poor condition, with large holes, which may have impeded them providing physical protection from mosquitoes [47]. We also noted that most respondents mentioned having washed their nets using detergent and then drying them directly in the sun. According to Morris et al. [48], direct sunlight is harmful to pyrethroid-based insecticides because ultraviolet rays break down pyrethrin molecules, thereby rendering the insecticide ineffective. However, the role played by sunlight in enhancing or rendering pyrethrin ineffective is still controversial. Nevertheless, bio-efficacy tests showed that eight of the ten bed nets tested were still optimally effective (≥ 80% mortality) against the susceptible laboratory strain of *An. gambiae* (reference susceptible Kisumu strain). Therefore, in addition to their physical degradation, the loss of efficacy of the LLINs was more likely due to increased insecticide resistance in local vectors. None of the nets tested was effective against the wild *An. gambiae* (*s.l.*) strain, which exhibited a high level of resistance to the pesticides deltamethrin and permethrin used to impregnate the nets. The source of this high level of resistance may be the *kdr* mechanism as the L1014F mutation was observed in high frequency in *An. gambiae* (*s.l.*). These findings are consistent with those reported in studies that have been conducted across the country and which indicate a rapid evolution of insecticide resistance in malaria vector populations [49, 50]. However, other mechanisms, such as metabolic resistance, could also be involved [49, 51, 52]. It is worth mentioning that Mvoua is a village mainly inhabited by farmers. The locality is characterized by the presence of many banana plantations and cocoa trees, with a consistently high use of pesticides. As reported by Kerah-Hinzoumbé et al. [53], a mosquito population could generate resistance to pyrethroids through exposure in areas where there is an extensive use of pesticides in agriculture.

## Conclusion

The findings of this study highlight high and perennial malaria transmission in Mvoua, with the highest infection rate in *Anopheles* vectors observed in the short dry season. Three major vectors, namely *An. funestus*,* An. coluzzii* and *An. gambiae*, were responsible for the transmission of the disease. This vector displayed a high level of resistance to pyrethroid insecticides due to the *kdr* mutation, but also probably due to metabolic mechanisms. Although there was a high level of LLIN possession among the households, universal coverage was not reached and LLINs found in the locality were no longer effective against the local *An. gambiae* (*s.l.*) strain. The National Malaria Control Program should provide the locality with new LLLNs every 3 years, as recommended by WHO. Preferably, to manage the high pyrethroid resistance observed, these LLNs should incorporate a synergist to block the action of detoxification enzymes. People should be educated on the use and maintenance of LLINs, with an emphasis on the importance of sleeping under LLINs irrespective of season, as well as on the damaging impact of detergents used for their washing.

## Data Availability

The datasets generated and/or analysed during the current study are available from the corresponding author on reasonable request.

## Abbreviations

DDT: Dichlorodiphenyltrichloroethane
EIR: Entomological inoculation rate
ELISA CSP: Enzyme-linked immunosorbent assay to detect the circumsporozoite protein
HLC: Human landing catch method
*kdr*: Knockdown resistance
LLIN: Long-lasting insecticidal net
pHI: Proportionate hole index
WHOPES: World Health Organization Pesticides Evaluation Scheme
